# The Value of Endoscopic Exposure of Round Window in Cochlear Implant via Facial Recess

**DOI:** 10.1055/s-0043-1775811

**Published:** 2024-02-05

**Authors:** Mena Maher Nassif, Islam Mohamed Hussein Darahem, Ahmed Abdelmoneim Teaima, Mustafa Mohamed Mustafa, Mohamed Saad Hassab Allah, Samer Ahmed Ibrahim

**Affiliations:** 1 Faculty of Medicine, Ain Shams University, Cairo, Eygpt

**Keywords:** endoscopic-assisted CI, round window, endoscopic ear surgery

## Abstract

**Introduction** Cochlear implantation has been considered as the best treatment in patients with severe to profound hearing loss unaidable with hearing aids. The main value of endoscope-assisted cochlear implantation is improved visibility of the RW

**Objective** to assess the value of endoscopic assisted CI surgery via facial recess approach without elevating tympanic anulus.

**Methods** This Prospective case series study non-randomized sample was performed on 50 patients with severe to profound hearing loss unaidable with hearing aids undergoing unilateral endoscopic assisted cochlear implant surgery with round window electrode insertion

**Results** There were 23 male and 27 female patients. Most of the cases were children (41 cases). Of those 50 patients, 39 were prelingually hearing impaired. Four cases had various inner ear abnormalities. The standard mastoidectomy and Posterior Tympanotomy approach were used for all cases. Endoscopic identification of the RW through the PT enabled us to perform regular surgery in all cases. The current study concludes the difference between microscopic exposure and endoscopic exposure represented by Saint Tomas classification found that endoscopic exposure of round window classification is better represented by downgrading in the classification of round window exposure as type I 29(58%), type IIa 18(36%) type IIb 3 (6%) Non were type III by endoscopic exposure compared to microscopic exposure of round window is a type I 7(14%), type II 14(28%), type IIb 22(44%) and type III 7 (14%).

**Conclusion** Endoscopy proved a great value in exposure and identification of RW in CI surgery through posterior tympanotomy approach,

## Introduction

The temporal bone is a very complicated anatomical structure with many hidden areas that occur during postnatal growth, and all these could implicate CI surgery. Also, treatment of chronic otitis media with cholesteatoma was used to be achieved endoscopic. Recently endoscopic cochlear implantation surgery was also reported. Correct Identification of the round window during cochlear implant surgery is a must for successful electrode insertion in the cochlea for minimizing damage of residual hearing by insertion an endoscope through the facial recess and obtaining a clear exposure of the round window membrane, adjusted by the angled endoscopy and coming closer with endoscopy showing the small blood vessels on the tegmen of the round window niche when it removed full detailed view of round window membrane obtained. Many developmental factors contribute to RW position and exposure of round window membrane, the anterolateral displaced facial nerve always considered the limiting point for appropriate wide facial recess. Optimal electrode position has been considered a challenging point beside correct insertion of the electrode through round window membrane and minimizing the residual hearing loss even with bony cochleostomy which isn't desirable. We aimed in the study to assess value of endoscopic aided CI surgery via facial recess approach without elevating tympanic anulus.

## Methods

Case Series Study (Nonrandomized convenient sample) 50 patients with profound hearing loss, unaidable with hearing aids undergoing unilateral endoscopic assisted cochlear implant surgery with round window electrode insertion. Ethical Committee approval was received from Ethical Committee of Ain shams University, Faculty of Medicine (MD/93/2020). Written consent was obtained from all participants who participate in this study. Statistical analysis: The saved data was studied, implied, arranged, and presented to a PC using IBM© SPSS© Statistics version 25 (IBM© Corp., Armonk, NY) and JMP® version 13.2.1 (SAS© Institute Inc., Cary, NC).

**Fig. 1:**
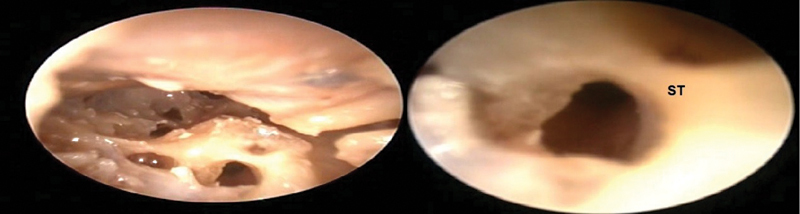
Pie chart shows the difference in classification between the microscopic exposure and endoscopic exposure.

There were Fifty endoscopic assisted cochlear implants surgery was done and prospectively evaluated, around 23 boy (46%) and 27 girl (54%) patients. The most of them were children 41 (82%). Of those 50 patients, 39 (78%) were prelingually hearing impaired. Four cases (8%) with many inner ear anomalies. The classical mastoidectomy and Posterior Tympanotomy approach were used for all cases. Med-El (FLEX28™; FLEX24™ Med-El, Innsbruck, Australia), Advanced Bionics (AB)Mid-Scala Electrode and Cochlear™ (NUCLEUS® CI422 WITH SLIM STRAIGHT, NUCLEUS® CI24RE CONTOUR ADVANCE™, Sydney, Australia) implants were used. Endoscope-aided exposure of the RW membrane through the posterior tympanotomy was performed in all patients. Rigid endoscopes with 0° and 30° degrees with variable width (1.9, 2.7 and 4 mm) and a high-definition camera system (Karl Storz, Germany) were used for the endoscopic evaluation. The RW membrane was identified by inserting the endoscope through the posterior tympanotomy without elevating the tympanic annulus. Then we switched to the microscope and drilled the round window niche using both hands until a circumferential panorama of the round window membrane is achieved. After appropriate exposure, the electrode was introduced through an opening in the round window membrane mostly under microscopical vision. Endoscopic identification of the round window through the posterior tympanotomy qualified us to perform accepted surgery in all patients. Also, endoscopic cochleostomy was tried in few cases. We did many Cadaveric dissection for optimal training for RW and ST identification translated as a successful identification in patients underwent endoscopic CI surgery ( Figure 1 ).

## Results

Among the fifty-patient included 23 (46%) are male patients and 27 (54%) are female. Most of the patients were prelingual less than 5 years 41 (82%) ( Table 1 ).

**Table 1:** Show the demographic data of the patients including age and gender

		No	%
Gender	F	27	54.0%
	M	23	46.0%
Age	<5	41	82.0%
	>5	9	18.0%

Radiological evaluation of all patients included most of the patients are normal finding 44(88%), two cases (4%) FN anterior displaced, one case (2%) IP II, one case (2%) Wide vestibular aqueduct, one case (2%) osteogenesis imperfecta, and one case (2%) post-traumatic ( Table 2 ).

**Table 2:** Show the radiological finding of the included patients

		No	%
Radiological finding	Facial n anterior	2	4.0%
	IP II	1	2.0%
	Normal	44	88.0%
	Osteogenesis imperfecta	1	2.0%
	Post traumatic	1	2.0%
	Wide vestibular aqueduct	1	2.0%

Most of our cases underwent RW electrode insertion 43 (86%) and only seven cases after endoscopic evaluation underwent bony cochleostomy. Intraoperative finding of facial nerve course nearly normal in 46 (92%) between all cases while other cases showed displaced anterior facial nerve course translated as narrow PT. After Endoscopic examination through PT, we found 42 (84%) cases with normal cochlear orientation while other cases presented with cochlear rotation:8 cases (16%) among medial. posterior, posteromedial, and anteromedial rotation of the cochlea. Many factors contribute to the width of PT including mainly the position of the Facial nerve. Among all cases included least width of PT to introduce the endoscopy was 1.9 mm while the average was between 2 to 3 mm diameter measured roughly by the width of different endoscopes ( Table 3 ).

**Table 3:** Shows the course of FN, operative approach, and width of PT

		No	%
Chochleostomy RW or ERW	Bony cochleostomy	7	14.0%
	Round window	43	86.0%
FN course	Abnormal	4	8.0%
	Normal	46	92.0%
Cochlea rotation	Antero medial	1	2.0%
	Medial	2	4
	Normal	42	84.0%
	Posterior	2	4.0%
	Postero medial	3	6.0%
Posterior_Tympanotomy_width_mm (1.9 to 3 mm) _	1.90	1	2.0%
	2.00	1	2.0%
	2.20	1	2.0%
	2.30	37	74.0%
	2.70	9	18.0%
	3.00	1	2.0%

**Fig. 2:**
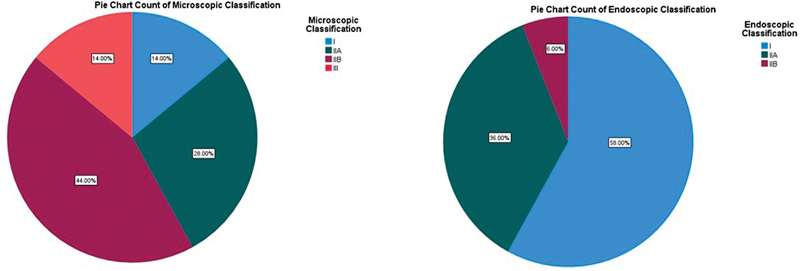
Rigid 0 Degree 1.9mm otoendoscopy (The best to use according to our study).

Pie Chart ( Figure 2 ) shows the difference between microscopic exposure and endoscopic exposure according to the St Tomas classification regarding round window exposure. Endoscopic exposure of round window was type I in 29 (58%), type IIa in 18(36%) type IIb 3 in (6%). Non were type III by endoscopic exposure. This is in comparison to microscopic exposure of round window which was type I in 7(14%), type IIa in 14(28%), type IIb in 22(44%) and type III in 7 (14%).

**Fig. 3:**
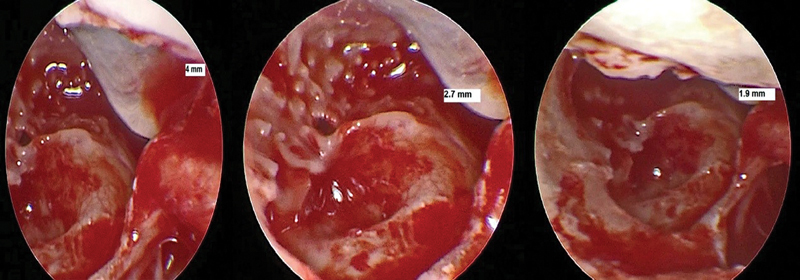
exposure of round window in different cases of bad quality and unclear identification with using low quality video recording system.

For better quality images and wider field, Rigid endoscopes with 0° and 30° degree with variable width (1.9, 2.7 and 4 mm) and a high-definition camera system were used for the endoscopic evaluation and was used to evaluate the round window ( Figure 3 ). It was found that using a rigid, 0°degree, 1.9mm diameter endoscopy inserted via posterior tympanotomy was the best of all sizes and angles in improving the optics and magnification, providing a better-quality image and a comprehensive field of view ( Table 4 ).

**Table 4:** Shows the value of endoscopic use (According to Thomas Hospital Classification)

	Microscopic Classification		Endoscopic Classification	
	No	%	No	%
I	7	14%	29	58%
IIA	14	28%	18	36%
IIB	22	44%	3	6%
III	7	14%	0	0.0%

Note the lack of invisible RW with endoscopic-assisted approach. Note also that about 60% of RW showed as Type I with endoscope assistance, as compared to less than 15% with microscope alone.

The anterolateral displaced facial nerve always considered the limiting point for appropriate wide facial recess and incomplete identification of the round window.

## Discussion

**Fig. 4:**
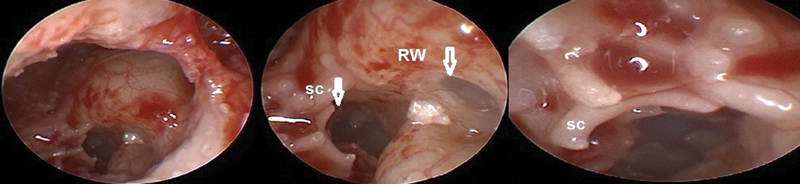
Endoscopic view of round window using 1.9mm,0-degree endoscopy. (SC) subcochlear canaliculus (commonly mistaken for the true RW); (RW)Round window (left ear).

**Fig. 5:**
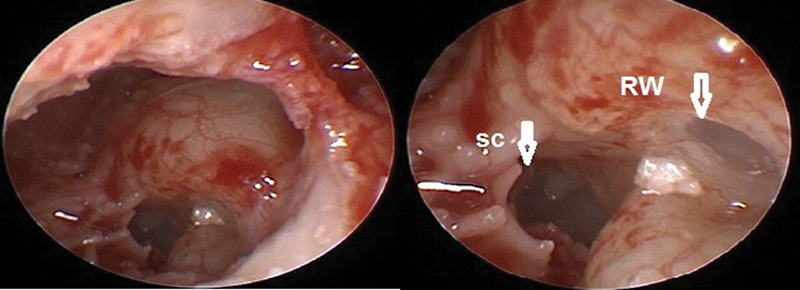
Identification of RW membrane (left ear).

**Fig. 6:**
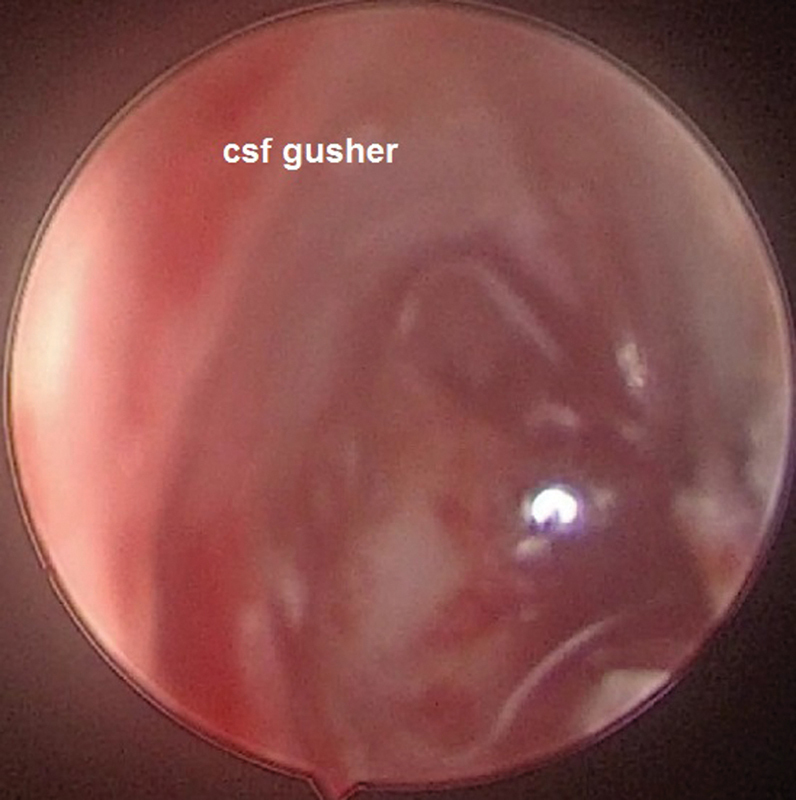
(Left ear) CSF gusher (dot of light reflection) in case IP II Endoscopic view RW wasn't clearly identified.

Patients complaining of profound hearing loss unaidable with hearing aids were concerned of cochlear implantation (CI) as the most suitable treatment. 1 A posterior tympanotomy approach used for cochlear implantation as a standard approach 2 with electrode insertion either RW insertion or bony cochleostomy. 3 Identification of the round window membrane through the facial recess is a must during cochlea implantation, whatever approach the surgeon uses for insertion (RW or cochleostomy). 4 The facial recess is a triangular area bounded by the fossa incudis superiorly, a vertical segment of the facial nerve medially, and the chorda tympani laterally and provides safe access to the basal turn through the round window niche. The anterolateral displaced facial nerve always considered the limiting point for appropriate wide facial recess even with experienced surgeons that require using small diameter endoscopy 1.9mm,0degree thought posterior tympanotomy to give panoramic very clear identification of round window to be sure about the exact site of the round window. 5 The round window niche is considered one of the covered areas that the endoscope can precisely recognize and discriminate it from the hypotympanic structure and Subcochlear Canaliculus ( Figure 4 ). These pros help to decrease complications and allow atraumatic electrode insertion in the scala tympani for hearing conservation. cases with the normal position of facial nerve predicted by preoperative CT give the surgeon the suitable width of PT for introducing larger diameter of endoscopy 2.7mm 0-degree endoscopy or even 4 mm endoscopy that could be used at the edge of PT not introduced through it, providing a brighter image ( Figure 5 ). The profit of endoscope-aided cochlear implantation is upgraded visibility of the round window and lowered the risk of complications, especially in patients with congenital deformities like IP II ( Figure 6 ).

This study presented a better solution by insertion an endoscope through the facial recess and obtaining a clear exposure of the round window membrane, adjusted by the angled endoscopy and coming closer with endoscopy showing the small blood vessels on the tegmen of the round window niche when it removed full detailed view of round window membrane obtained. and the aim not only performing membranous cochleostomy through RW membrane, but also optimal electrode position has been considered a challenging point beside correct insertion of the electrode and minimizing the residual hearing loss. 6 Bony cochleostomy which is more undesirable could be used when there is limited access. 7

In the study, we focused to assess value of endoscopic aided CI surgery via facial recess approach **without elevating tympanic anulus** . This prospective study was conducted including 50 patients from April 2020 till March 2022. All patients presented with severe to profound SNHL undergoing CI via trans- mastoid posterior tympanotomy approach.

**Fig. 7:**
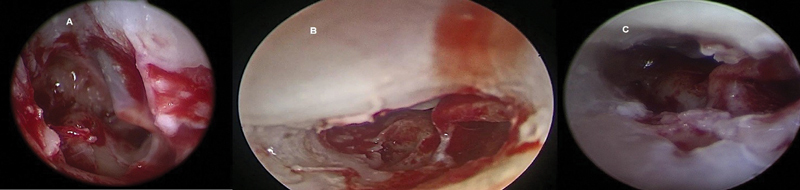
Identification of RW membrane (Narrow PT) using rigid 0-degree 2.7 mm endoscopy **(A)** right ear and ( **B** ) ( **C** ) left ear.

**Fig. 8:**
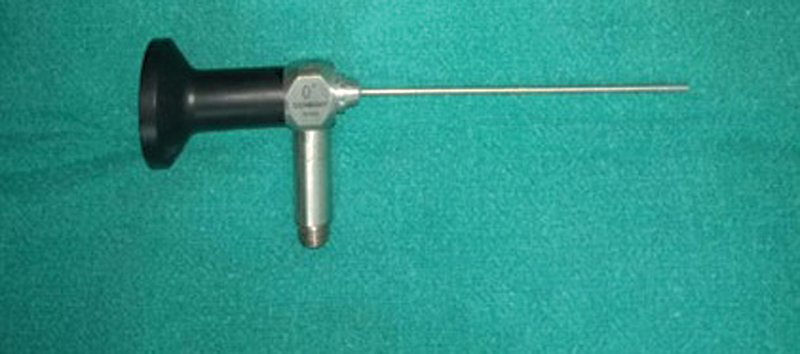
Endoscopic Identifaction of RW and SC (Subcochlear canaliculs).

**Fig. 9:**
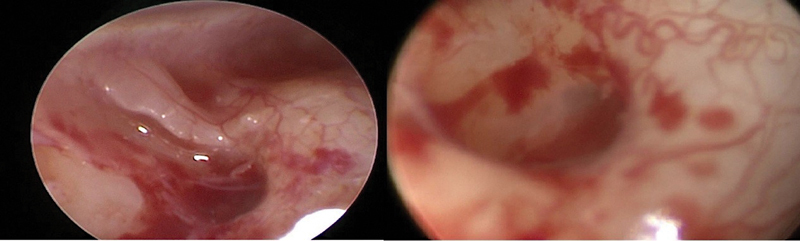
Endoscopic view of RW and Scala Tympani (ST) in cadaveric dissection (right ear).

**Fig. 10:**
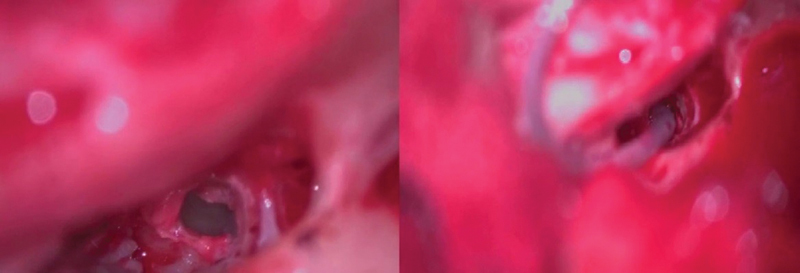
Microscopic view of ST before and after electrode full insertion (left ear).

**Balkany** was the first to use a fiberoptic otologic endoscope during cochlear implantation help in identification of normal the cochlea structures. 8 The use of a rigid endoscope to completely visualize the RW region from posterior tympanotomy has been reported recently with the use of 0° degree 2.7 mm width, 0° and 30° degree 2.7mm and 4mm width. 9 Endoscopes of 2.7 mm and 4mm diameter probably cannot enter a standard posterior tympanotomy and there is still suboptimal orientation, with a standard posterior tympanotomy of 2 mm ( Figure 7 ). With introduction of a 1.9 mm outer diameter endoscope ( Figure 8 ), the RW niche is almost always completely visible so that the thinning of the lips of the RW niche under endoscopic view can be done, until the projection of the lateral wall of the Scala Tympani (ST) ( Figure 9 ). Thus, avoids injury to functionally important structures through removing sufficient bone of the crista fenestrae to get positioned access to the scala tympani this allows the soft introduction of the electrode into the basal turn ( Figure 10 ). This approach poses many advantages in cases of particularly curved external auditory canal or small facial recess, avoiding over-thinning of the posterior wall with potential breakdown, and potential facial damage especially in case of cochlear abnormality.

**Fig. 11:**
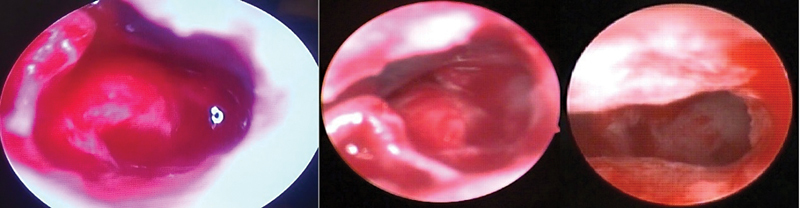
Endoscopic view of RW by three different diameter of Endoscopy showed different quality in the same patient (left ear).

Using bad types of video recording systems or otoendoscopy will get low quality images ( Figure 11 ) and not getting clear, bright and high-quality identification.

**Fig. 12:**
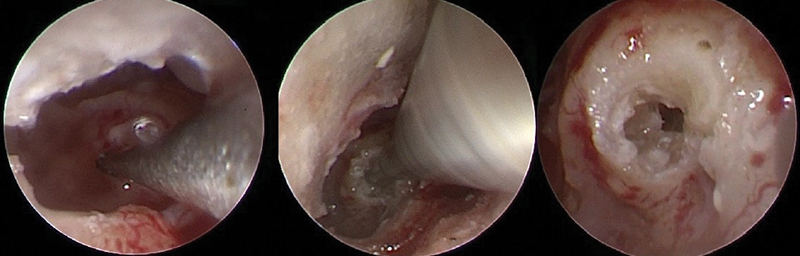
Endoscopic Cochleostomy using a rigid 0-degree 1.9 mm endoscopy (left ear). The main disadvantage was single handed procedure.

**Fig. 13:**
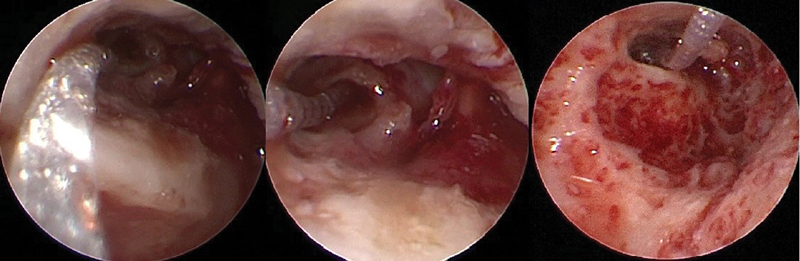
Endoscopic almost full electrode insertion using a rigid 0-degree 1.9 mm endoscopy (left ear).

The current research study revealed that endoscopy has a great value in the endoscopic exposure of round window in compared to microscopic exposure according to the St Tomas classification described by Jiang et al. Our study concludes the difference between microscopic exposure and endoscopic exposure represented by Saint Tomas classification regarding round window exposure found that endoscopic exposure of round window classification is better represented by downgrading in the classification of round window exposure as type I 29(58%), type IIa 18(36%) type IIb 3 (6%) Non were type III by endoscopic exposure compared to microscopic exposure of round window is a type I 7(14%), type II 14(28%), type IIb 22(44%) and type III 7 (14%). In addition, the electrode could be inserted under endoscopic view in some of our patients ( Figure 12 ). **However,** as some electrodes are malleable and springy, it will be challenging with holding endoscopy by one hand and insertion with the other ( Figure 13 ). We suggest that endoscopy will also have a great benefit in patients with otosclerosis obliterating the round window, Ossified Cochlea, and revision CI Cases. Recognition of the fustis, an anatomical landmark to the round window, allows precise positioning of cochleostomy. Additionally, the broader visual field provided by the endoscope decreases the necessity for a more extensive approach.

These findings agree with previous studies. **Güneri et al** 10 did a retrospective study of endoscopic-assisted cochlear implant and concluded that the endoscopy improved the exposure of the round window through the classical posterior tympanotomy approach and decrease the risk of complication or need to switch to other surgical approaches in case of difficult exposure. **Jain et al** 11 did a study to relate microscopic and endoscopic exposure of the round window membrane during cochlear implantation in 20 patients. Their results showed that using endoscopic aided cochlear implant improved the identification of round window and reduced the complications, especially in patients with cochlear malformations. Moreover, **Fouad et al** 12 had another retrospective study describing the role of endoscopy in round window identification in the cochlear implant in 13 patients, and showed that the endoscopy has a great role in the identification of round window in some difficult cases. 12 **Chen et al** 9 conducted another study about using endoscopy in finding round window membrane in cochlear implant and decided that endoscopy is beneficial for locating it during cochlear implantation surgery, including surgeries for inner ear anomalies. **Nassif et al** 13 discussed the probability of a round window identification through the posterior tympanotomy using rigid endoscopy into eight patients undergoing cochlear implant and concluded that complete endoscopic exposure through the posterior tympanotomy allows identification of the round window niche as well as the direction of the scala tympani.

**Orhan et al**^14^ described the benefit of using the endoscopy in cochlear implantation in cases failed to get adequate round window exposure in three patients by using rigid 0-degree endoscope 2.7 mm wide after elevating the tympanomeatal flap. Then endoscopy pass through external auditory canal to expose round window and remove round window niche and insert the electrode with aid of endoscopy pass through external auditory canal. **Dia et al** 15 describe patient outcomes after transcanal endoscopic cochlear implantation and concluded that endoscopic cochlear implantation may become a practicable, harmless, and possible alternative to the standard open transmastoid approach. 15 However, going through the external canal caries many risks, and an endoscope passing through the posterior tympanotomy would avoid such transcanal exposure of the RW. This provides a safer and softer approach, in our opinion.

**The strength points** of this study are that it is prospective study design, availability of different diameters of otoendscopy, and a larger sample size compared to other studies. **The limitations** of the study are worthy of mention., This study didn't include enough inner ear malformation patients, Also, it didn't include revision patients, nor patients with impaired cochlear patency, where the endoscope is expected to play a very helpful role.

## Conclusion

Endoscopy proved a great value in exposure and identification of RW in CI surgery through the posterior tympanotomy approach, especially in difficult cases. Endoscopic assisted CI through facial recess could be easily done using 1.9 mm 0-degree endoscopy even in cases e narrow PT or rotated cochlea.
